# The mechanism of m6A modification in cardiovascular diseases: A systematic review

**DOI:** 10.1016/j.gendis.2025.101672

**Published:** 2025-05-05

**Authors:** Hongjiao Liu, Tao Song, Yan Huang

**Affiliations:** a Department of Cardiology and Cardiovascular Research Institute, Renmin Hospital of Wuhan University, Wuhan, Hubei 430060, China; b Hubei Key Laboratory of Cardiology, Wuhan, Hubei 430060, China

**Keywords:** Arrhythmia, Cardiovascular diseases, Coronary atherosclerotic heart disease, Heart failure, Ischemic reperfusion injury, N6-methyladenosine (m6A), Pulmonary hypertension

## Abstract

N6-methyladenosine (m6A) is the most prolific and conserved epigenetic modification of eukaryotic RNAs and is closely associated with the transcription, cleavage, translation, and degradation of target mRNAs. Cardiovascular disease (CVD) is the leading cause of death globally, with a significant research area focusing on understanding its pathogenesis and identifying effective therapeutic strategies. Recent advances in RNA methylation have revealed that m6A RNA modifications play a critical role in the initiation and progression of CVDs, potentially offering new insights into the development of these diseases. Interactions among various components influencing m6A modification levels regulate the effects of downstream targets, either by promoting or inhibiting CVD progression. This review connects the different types of CVDs and discusses the regulatory processes and intricate interactions between m6A methyltransferases and demethylases. We suggest that m6A RNA methylation could uncover potential targets for diagnosing and treating diseases, providing a clear view of how m6A modification affects CVDs and explaining the related molecular mechanisms and biological functions.

## Introduction

Cardiovascular disease (CVD), the leading cause of death globally, is linked to increased morbidity, highlighting the need to elucidate its pathogenesis and develop effective therapies.^1^^,^^2^ The risk factors for CVD are multifaceted and involve both genetic and non-genetic elements such as smoking, high cholesterol, and high blood pressure.^2^^,^^3^ In recent years, epigenetic research has provided new insights into various diseases. Recent studies have stressed the vital role of m6A RNA modification in both physiological and pathological conditions, particularly in the onset and progression of cancer, metabolic diseases, and CVDs.^4^

**Figure 1:**
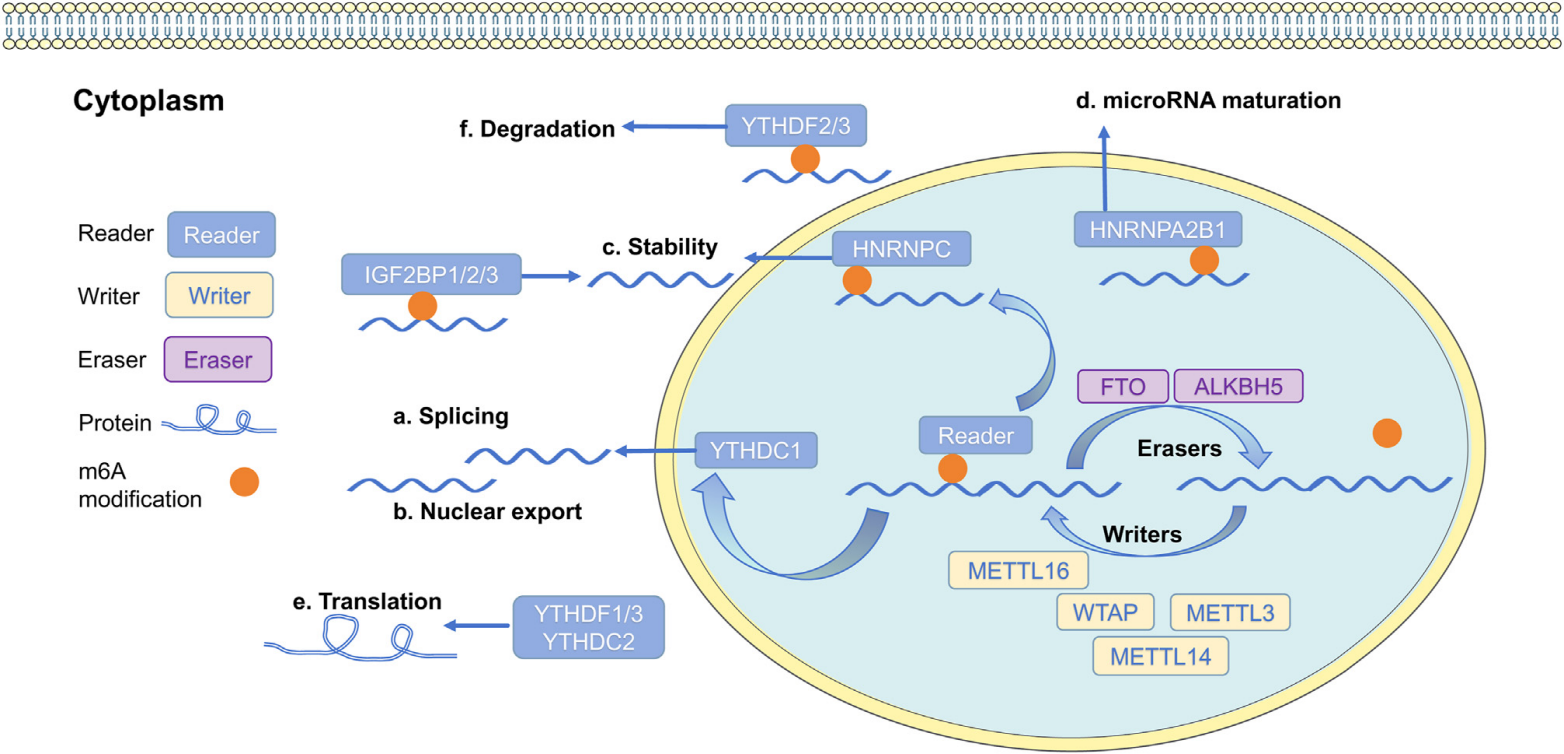
The molecular mechanism of m6A methylation. The m6A modification involves three key regulators: m6A methyltransferases (writers), m6A demethylases (erasers), and m6A-binding proteins (readers). METTL3, METTL14, WTAP, and METTL16 function as writers to install m6A methylation in this process. Demethylation is carried out by demethylases such as FTO and ALKBH5, while m6A reader proteins, including YTHDF1/2/3, IGF2BP1/2/3, and YTHDC1/2, recognize the modification. These reader proteins identify specific binding sites to regulate RNA processing, encompassing splicing, nuclear export, stability, microRNA maturation, translation, and degradation. m6A, N6-methyladenosine; METTL3, methyltransferase like 3; METTL14, methyltransferase like 14; METTL16, methyltransferase like 16; WTAP, Wilms tumor 1-associating protein; FTO, Fat mass and obesity-associated protein; ALKBH5, Alk B homolog 5; YTHDF1/2/3, YTH N6-methyladenosine RNA binding protein F1/2/3; YTHDC1/2, YTH N6-methyladenosine RNA binding protein C1/2; IGF2BP1/2/3, insulin-like growth factor 2 mRNA binding protein 1/2/3; HNRNPA2B1, Heterogeneous nuclear ribonucleoprotein A2/B1; HNRNPC, Heterogeneous nuclear ribonucleoprotein C.

Epigenetic modifications act as bridges between genotypes and phenotypes, regulating heritable gene expression without altering the DNA sequence. This process involves methylation, histone modification, chromatin remodeling, and non-coding RNA regulation. At the post-transcriptional level, RNA modification is the most common type of reversible modification and can regulate gene expression by controlling RNA metabolism and related pathways.^4^^,^^5^ To date, over 170 RNA modifications have been identified in both non-coding RNA and mRNA, including N7-methylguanosine (m7G), N1-methyladenosine (m1A), N6-methyladenosine (m6A), 5-hydroxymethylcytosine (hm5C), 2′-O-methylation (Nm), and N4-acetylcytidine (ac4C).^6^, ^7^, ^8^, ^9^, ^10^, ^11^, ^12^, ^13^, ^14^, ^15^ Among these modifications, m6A is enriched near stop codons or in the 3′ untranslated regions (3′ UTRs). This is the first confirmed and the most abundant internal modification.^16^^,^^17^ The m6A modification process is highly dynamic and reversible, regulated by a balance among three homologous factors: m6A methyltransferases (“writers”), m6A demethylases (“erasers”), and m6A-binding proteins (“readers”), as illustrated in Figure 1. During this process, m6A methylation is primarily installed by methyltransferases, such as methyltransferase-like 3 (METTL3), methyltransferase-like 14 (METTL14), Wilms' tumor 1-associating protein (WTAP), RNA-binding motif protein 15, vir-like m6A methyltransferase associated, zinc finger CCCH-type containing 13, methyltransferase-like 5 (METTL5), and zinc finger CCHC domain-containing protein 4 (ZCCHC4), and methyltransferase like 16. Demethylases, such as fat mass and obesity-associated protein (FTO) and AlkB homolog 5 (ALKBH5), carry out demethylation. The recognition of the modification occurs via m6A reader proteins, including YTH N6-methyladenosine RNA-binding proteins 1/2/3 (YTHDF1/2/3), eukaryotic translation initiation factor 3 subunit A, and YTH N6-methyladenosine RNA-binding proteins C1/2 (YTHDC1/2), which identify specific binding sites to perform multiple biological functions, including structural stabilization, translation, and RNA degradation.^18^ The writing and erasing of m6A mainly occur during transcription with regulators such as the “writers”, “erasers”, and the “reader” complex mainly located in the nucleus. After exporting the marked mRNA to the cytoplasm, m6A binds specifically to readers, thereby regulating the structural stability, translation, and specific localization of mRNAs.^19^^,^^20^ The function of m6A is complex and is primarily related to its spatial and temporal distribution. For instance, m6A modification in the 5′ cap region and 3′ poly (A) region of mRNA contributes to maintaining mRNA stability, alternative splicing, extracellular transport, and translation. Furthermore, m6A plays distinct regulatory roles in different RNA species (Table 1). m6A modulates precursor miRNA processing and downstream target regulation, thereby affecting endothelial cell proliferation and atherosclerosis.^21^^,^^22^ In long non-coding RNA (lncRNA), m6A modifications regulate the structural stability and interactions with chromatin-remodeling proteins, mediating myocardial regeneration and pathological hypertrophy.^23^ tRNA m6A modifications affect translational fidelity and abundance, indirectly influencing protein synthesis during cardiac stress.^24^ Within rRNA, m6A ensures ribosomal maturation and function, with METTL5/ZCCHC4-mediated 18S/28S rRNA methylation preserving translational accuracy in terminally differentiated cardiomyocytes.^25^^,^^26^ For circRNAs, m6A facilitates biogenesis and nuclear export, enabling their roles as miRNA sponges and regulators of vascular remodeling.^27^^,^^28^ These epitranscriptomic mechanisms highlight m6A's context-dependent roles in CVDs and offer therapeutic targets for RNA-specific dysregulation.

**Table 1:** The modulation of different RNA types by m6A regulators.

RNA Type	Role of m6A Modifications	Modification factors: Writers, Readers, or Erasers
mRNA	Regulate mRNA stability, translation efficiency, and processing.^19^^,^^20^ Modulate mRNA degradation via m6A reader proteins.^4^^,^^29^ Influence alternative splicing and transport.^30^	Writer: METTL3/14/16^19^^,^^20^ Reader: YTHDC1/2, YTHDF1/2/3, HNRNPC/G, HNRNPA2B1^4^^,^^19^^,^^20^^,^^29^ Eraser: FTO, ALKBH5^19^^,^^20^
miRNA	Affect the processing of precursor miRNAs (pre-miRNAs) to mature miRNAs.^21^ Modulate miRNA expression and their downstream target genes.^22^ Impact cell proliferation, migration, and apoptosis through regulation of specific miRNAs.^22^	Reader: YTHDF2^21^ Eraser: FTO^22^
lncRNA	Change the structure of lncRNA and regulate lncRNA stability or degradation^31^ Affect its interaction with proteins and mediate gene transcription repression.^32^ Play a regulatory role in the process of myocardial regeneration in the neonatal mouse.^23^	Writer: METTL3^23^^,^^32^ Reader: YTHDF2, YTHDC1, HNRNPC^31^^,^^32^
tRNA	Associated with regulating tRNA abundance and function, impacting protein synthesis efficiency and fidelity.^24^	
rRNA	Involve in rRNA maturation and processing, impacting ribosome assembly and function.^25^^,^^26^ Enhance the efficiency of protein synthesis by modulating ribosomal function and stability.^25^^,^^26^ Linked to regulation of gene expression and fatty acid metabolism.^33^^,^^34^	Writer: ZCCHC4, METTL5/16^25^^,^^26^^,^^33^^,^^34^
circRNA	Affect the biogenesis of circRNAs from pre-mRNA and help circRNA nuclear export. Influence the stability and function of circRNAs, which regulate gene expression and cellular function. Associated with various diseases, including various cancers and neurological disorders.^27^	Writer: METTL3^27^ Reader: IGF2BP1, YTHDC1^27^ Eraser: ALKBH5^27^

The dynamic modification of m6A methylation in different tissue cells affects life processes by influencing downstream molecules, immune responses, and stem cell renewal, potentially leading to diseases or pathological states.^35^ Berulava et al identified more than 403 m6A methylation targets in mouse and human heart tissue, suggesting that m6A methylation may be involved in the early stages of CVDs.^36^ Moreover, m6A plays a crucial role in maintaining calcium homeostasis and regulating circadian rhythms, autophagy, lipid metabolism, and inflammatory responses, all of which may contribute to the development of CVDs.^37^, ^38^, ^39^

Therefore, exploring the roles of RNA modifications in CVDs may enhance our understanding of the molecular mechanisms involved and aid in the development of novel biomarkers and therapeutic targets for CVD treatment.^37^^,^^40^^,^^41^ This review summarizes the mechanisms of m6A RNA methylation and related regulatory factors involved in this process. It discusses the roles of m6A methylation in coronary atherosclerotic heart disease, hypertension and pulmonary hypertension (PH), arrhythmia (AH), heart failure (HF), and cardiac reperfusion injury, concluding with their treatment prospects.

## m6A in coronary atherosclerotic heart disease

Coronary artery disease (CAD), characterized by chronic inflammation within the arterial walls, is considered one of the leading causes of death worldwide, primarily due to atherosclerosis.^42^ Atherosclerotic plaque formation is one of the pathological features of atherosclerosis.^43^ Chronic inflammation damages the arterial wall, leading to intimal hyperplasia, which is a pathophysiological response to acute or chronic sources of vascular damage. In conjunction with dyslipidemia, this process plays a crucial role in the development of CADs.^44^ The m6A modification has been shown to play an important role through its “writer”, “eraser”, and “reader” functions.^45^ Wu et al demonstrated that m6A methylation levels were significantly reduced in leukocytes from patients with atherosclerosis and in mouse models of the disease.^46^ Additionally, Quiles-Jiménez et al reported dysregulation of m6A modification in tissues from patients with carotid atherosclerosis, indicating that reduced levels of m6A modification were associated with decreased expression of “writers”, such as ZCCHC4 and METTL5, in early atherosclerotic lesion samples. In contrast, advanced lesions exhibited significant increases in the levels of WTAP and METTL3 (writers), eukaryotic translation initiation factor 3 subunit A and YTHDF2 (readers), and FTO (eraser).^47^ These findings preliminarily demonstrated the significant role of m6A modifications in CAD development.

Excessive proliferation and migration of vascular smooth muscle cells along with extracellular matrix synthesis are key events that contribute to the development of CADs.^48^, ^49^, ^50^, ^51^, ^52^ Zhang et al found that the overexpression of METTL14 in atherosclerotic vascular endothelial cells led to higher levels of m6A methylation, which suppressed pre-miR-19a and promoted the expression of miR-19a and DGCR8. These genes are involved in the proliferation and invasion of endothelial cells in the atherosclerotic vessels.^53^ Furthermore, Zhu et al showed that WTAP levels were increased in balloon catheter-injured rat carotid arteries, where WTAP regulated p16 through m6A modification. The WTAP/p16 axis affected the viability, proliferation, and migration potential of vascular smooth muscle cells induced by total Panax notoginseng saponins, highlighting the critical role of m6A modification in intimal hyperplasia.^54^ These studies suggest that the METTL14/m6A/miR-19a signaling pathway and/or WTAP-related m6A modifications may be potential therapeutic targets for CADs.

Dyslipidemia and abnormal lipid metabolism are significant contributors to CADs. High oxidized-low-density lipoprotein (ox-LDL) levels are an important risk factor for CADs.^55^ The size and thickness of carotid plaques have been found to be negatively correlated with leukocyte m6A levels, suggesting that decreased m6A levels in endothelial cells and leukocytes may serve as sensitive biomarkers for CAD progression.^46^^,^^56^ Recently, FTO has been shown to influence obesity and mitochondrial function in adipocyte precursors.^57^ FTO regulates m6A levels around splicing sites, affecting FTO-related exon splicing of the adipogenic regulator RUNX1 partner transcriptional co-repressor 1, which in turn influences the RNA-binding ability of serine- and arginine-rich splicing factor 2 and adipocyte differentiation. This indicates that FTO-dependent m6A demethylation is critical for regulating adipogenesis.^58^ Moreover, the up-regulated FTO reduces the expression of related genes, such as Iroquois-related homeobox 3 and Iroquois-related homeobox 5 via AT-rich interaction domain 5B. Silencing FTO results in elevated m6A methylation levels in the mRNAs of autophagy-related 5 and 7, as identified by YTHDF2, leading to mRNA degradation and reduced adipogenesis.^4^^,^^29^ However, the association between FTO and CADs remains unclear and requires further investigation.

**Figure 2:**
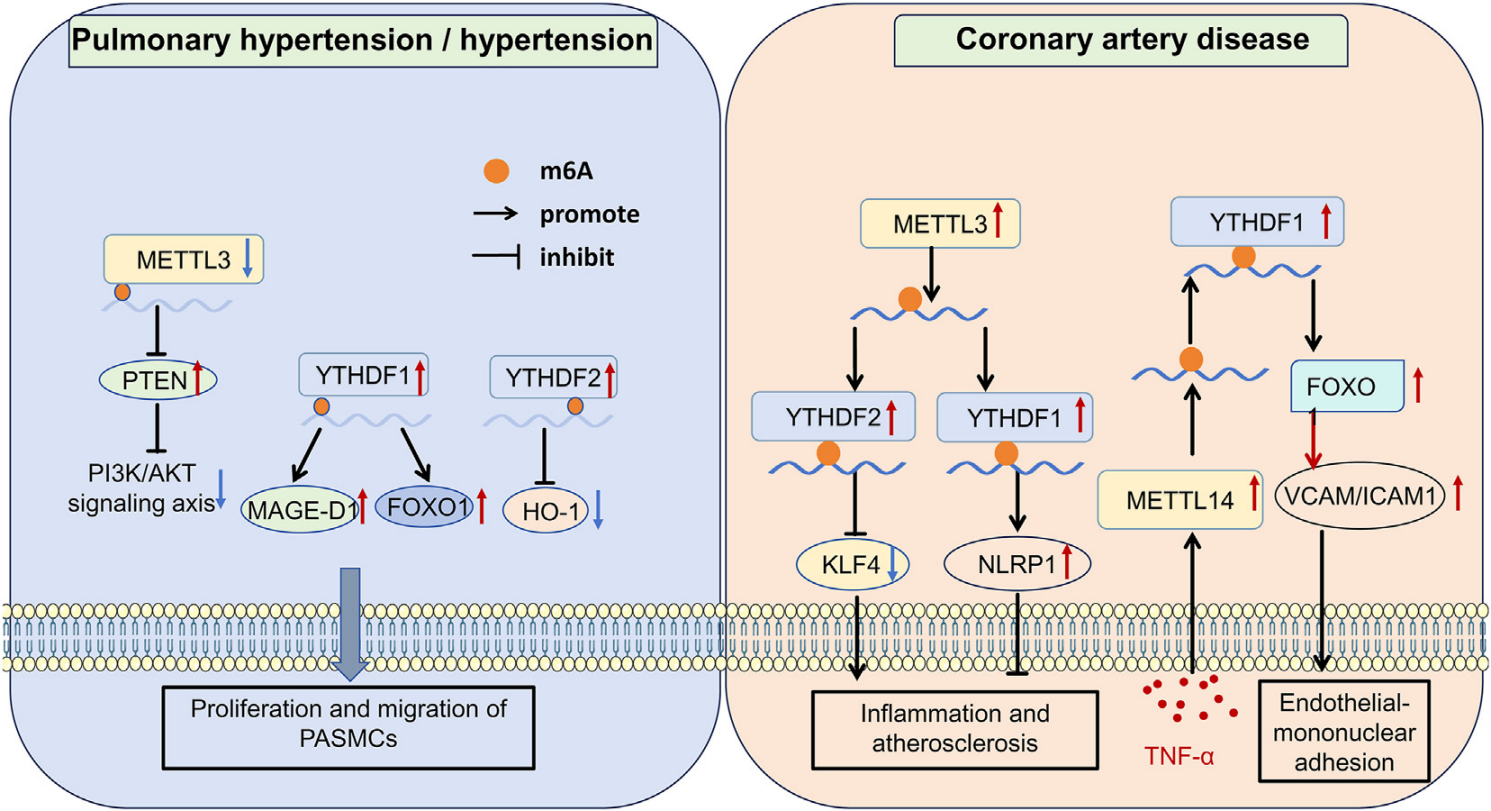
The molecular mechanism of CAD and PH. In CAD, the expression level of METTL14 is increased in TNF-α-induced endothelial cell inflammation, promoting FOXO1 mRNA translation through YTHDF1 recognition and leading to increased expression of ICAM1 and VCAM, thereby facilitating endothelial-mononuclear adhesion. The expression level of METTL3 is also elevated, down-regulating the expression of KLF4 via YTHDF2 recognition and up-regulating NLRP1 through YTHDF1 recognition, associated with inflammation. In PH and systemic hypertension, YTHDF1, YTHDF2, and METTL3 expression levels are increased. The overexpression of YTHDF1 up-regulates the expression of FOXO1 and MAGE-D1, promoting the proliferation and migration of pulmonary artery smooth muscle cells. METTL3 reduces PTEN expression, which up-regulates the PI3K/AKT signaling axis, contributing to the progression of hypertension. CAD, coronary artery disease; PH, pulmonary hypertension; TNF-α, tumor necrosis factor alpha; FOXO1, forkhead box O1; ICAM1, intercellular cell adhesion molecule-1; VCAM, vascular cell adhesion molecule; KLF4, Krüppel-like factor 4; NLRP1, nucleotide-binding oligomerization domain, leucine-rich repeat and pyrin domain-containing 1; MAGE-D1, melanoma-associated antigen D1; HO-1, heme oxygenase 1; PI3K, phosphoinositide 3-kinase; AKT, protein kinase B.

Inflammation also plays an important role in this process (Fig. 2). METTL3 has been suggested to function as a vascular endothelial response center for hemodynamic and atherosclerotic stimuli.^59^ Chien et al have identified METTL3 as a critical factor in monocyte adhesion and the atherogenic process within endothelial cells, which may be induced by oscillatory stress. This ultimately leads to hypermethylation, affecting two downstream targets, NLR family pyrin domain-containing 1 and KLF transcription factor 4, via YTHDF1 and YTHDF2. Consistent with these findings, both METTL3 and NLR family pyrin domain-containing 1 were up-regulated, while KLF transcription factor 4 was down-regulated in an atherosclerosis model. Knockdown of METTL3 appeared to prevent the atherosclerotic process, resulting in opposing effects on NLR family pyrin domain-containing 3 and KLF transcription factor 4. This suggests that METTL3-dependent m6A RNA modification may mediate an atherogenic inflammatory cascade in vascular endothelial cells.^59^ Recent evidence indicates that macrophages play a pivotal role in the pathogenesis of atherosclerosis, particularly in the initiation and perpetuation of inflammatory processes and the formation of atherosclerotic plaques.^60^ The expression of METTL3 in macrophages has been shown to increase with the progression of atherosclerosis, and the overexpression of METTL3 in peritoneal macrophages appears to elevate the levels of inflammatory factors and exacerbate inflammatory responses.^61^ Knockdown of METTL3 in myeloid cells *in vivo* seemed to reduce BRAF protein expression and attenuate ox-LDL-mediated phosphorylation of extracellular regulated protein kinases and phosphorylation of p38, which may negatively affect the progression of atherosclerosis and inflammatory responses. Sun et al indicated that upon ox-LDL stimulation, the expression of methyltransferases METTL3 and METTL14 in macrophages increased, whereas the total m6A modification level and the expression of Matrin-3, an RNA-binding protein, decreased, implying complex interactions among these regulators. In ox-LDL-stimulated macrophages and peripheral blood-derived monocytes from patients with CADs, the overexpression of Matrin-3 significantly appeared to attenuate the development of atherosclerosis *in vivo*, suggesting that Matrin-3 may represent a potential target for the treatment of CADs.^62^ Additionally, the relationship between METTL3 and M1 macrophage polarization may play a role in HF through the regulation of inflammation, as discussed in the chapter on CADs.^63^ Furthermore, METTL14 knockout significantly inhibited endothelial inflammation and reduced atherosclerotic plaque formation, indicating its possible therapeutic potential in managing atherosclerosis. Jian et al indicated that the expression level of METTL14 was significantly increased in TNF-α-induced endothelial cell inflammation. This up-regulation promoted forkhead box O1 mRNA translation through YTHDF1 recognition, subsequently enhancing the expression of intercellular adhesion molecule 1 and vascular cell adhesion molecule 1 via the direct action of the METTL14/forkhead box O1 complex on a related promoter.^38^

Moreover, m6A is thought to play a role in M1 macrophage polarization. For instance, the overexpression of METTL3 leads to m6A modification of STAT1 mRNA, thereby promoting M1 macrophage polarization. Conversely, the polarization of M1 macrophages can also increase the expression of METTL3, indicating a bidirectional interaction between the two. Therefore, METTL3 and METTL14 are potential targets for the treatment of excessive inflammatory responses and CADs.

## m6A and pulmonary hypertension (PH) and hypertension

PH is a pulmonary vascular disease characterized by progressive remodeling of the small pulmonary arteries, ultimately leading to right-sided HF. Pathologically, it is marked by irreversible vascular remodeling linked to the dysfunction of pulmonary artery smooth muscle cells, which relies on dynamic interactions among locally produced growth factors, vasoactive substances, and hemodynamic stimuli.^64^^,^^65^ Post-transcriptional modifications, particularly m6A modifications, are closely associated with the vascular remodeling observed in PH (Fig. 2).^45^^,^^66^^,^^67^ Hu et al indicated that in PH models, increased expression of YTHDF1 enhances the translation of melanoma-associated antigen D1 in an m6A-dependent manner, thereby promoting the proliferation, migration, and phenotypic switching of hypoxic pulmonary arterial smooth muscle cells (PASMCs). Inhibition of YTHDF1 appears to reverse this process, suggesting that targeting either YTHDF1 or melanoma-associated antigen D1 may represent a potential treatment strategy for PH.^68^ Furthermore, Kang et al demonstrated that YTHDF1 also regulates the translation of forkhead box protein M1 via m6A modification, which is associated with the proliferation of hypoxic PASMCs, and silencing of YTHDF1 may alleviate this process, consistent with the aforementioned results.^69^ Additionally, the down-regulation of FTO and the up-regulation of YTHDF1 in PH rat models may play critical roles in the pathogenesis of PH through modulating inflammation, glycolysis, the platelet-derived growth factor signaling pathway, transforming growth factor-β family receptor members, and ECM-receptor interactions.^39^

In addition to YTHDF1, YTHDF2 has been suggested to play a significant role in PH progression. During the early stages of PH, increased YTHDF2 levels in pulmonary macrophages may facilitate heme oxygenase 1 mRNA degradation in an m6A-dependent manner. The deficiency of YTHDF2 leads to altered macrophage phenotypes and inhibits PASMCs proliferation, potentially preventing Sugen5416/hypoxia-induced development of PH in mice.^70^ These findings suggest that the YTHDF2/heme oxygenase 1 pathway is a novel target for PH diagnosis and treatment.

METTL3 and METTL14 may also play essential roles in the development of PH. Studies conducted by Qin et al have demonstrated that elevated levels of METTL3 in hypoxic PASMCs may down-regulate the expression of phosphatase and tensin homolog in an m6A-dependent manner by forming the METTL3/YTHDF2/phosphatase and tensin homolog axis. This axis appears to enhance the phosphatidylinositol 3-kinase/serine–threonine kinase signaling pathway, thereby promoting the proliferation of PASMCs.^71^^,^^72^ Moreover, METTL3 may be involved in endothelial–mesenchymal transition, a principal pathological mechanism of PH.^73^ Through m6A methylation, METTL3 increases the level of transient receptor potential cation channel, subfamily C, member 6, promoting calcineurin/nuclear factor of activated T cells signaling and facilitating hypoxia-mediated endothelial-to-mesenchymal transition. Notably, knockdown of METTL3 could potentially reverse this transition.^74^ Additionally, Liu et al indicated that METTL14 down-regulates the expression of growth factor receptor-bound protein 2 related adaptor protein in collaboration with YTHDF2. Conversely, the overexpressing of growth factor receptor-bound protein 2 related adaptor protein has been shown to inhibit the Ras/ERK signaling pathway, alleviating the proliferation and invasion of PASMCs.^75^ In addition to its collaboration with YTHDF2 and growth factor receptor bound protein 2 related adaptor protein, SEDT2/METTL14-mediated m6A modification has also been demonstrated to influence the progression of PH.^76^ Collectively, these findings illustrate that METTL3 and its related pathways represent promising avenues for treating PH.^71^, ^72^, ^73^, ^74^, ^75^^,^^77^^,^^78^

Moreover, m6A has been shown to influence the circRNA-miRNA-mRNA co-expression network under hypoxic conditions.^77^ Zheng et al demonstrated that higher expression levels of collagen type IV alpha 1 chain and HNRNPA2B1 correspond to differentially expressed genes enriched in the regulatory axes related to muscle cell differentiation and vascular development pathways in PASMCs, as observed by single-cell RNA sequencing, and in patients with idiopathic pulmonary arterial hypertension. HNRNPA2B1 may regulate the proliferation and phenotypic transition of smooth muscle cells through the rno-miR-330p/TGFβR3 and rno-miR-125a-3p/slc39a1 pathways, which could impact PH development.^79^ Additionally, Zhang et al indicated that m6A modification may up-regulate the phosphorylation of eukaryotic initiation factor 2α, promoting the proliferation of PASMCs in monocrotaline-induced PH rats.^80^

Hypertension is a significant risk factor of cardiovascular, cerebrovascular, and renal diseases. It is a complex disorder that results from interactions between genetic and environmental factors.^81^ FTO is implicated in both PH and hypertension. A substantial body of evidence from recent studies indicates that the FTO rs9939609 variant may be a risk factor for hypertension, contingent on body mass index, in diverse populations.^82^, ^83^, ^84^ Moreover, the exposure to episodic chronic intermittent nicotine aerosols in pregnant rats has been shown to up-regulate FTO, resulting in reduced m6A levels in the vascular and NADPH oxidase 2 genes, which appears to enhance the angiotensin II-induced blood pressure response in male offspring. Dihydroartemisinin inhibits the FTO/nuclear receptor subfamily 4 group A member 3 axis, alleviating vascular smooth muscle cell proliferation and inflammatory responses stimulated by angiotensin II.^85^ In conclusion, although the specific mechanisms underlying these processes remain unclear, FTO and reduced m6A levels appear to be significant factors in hypertension progression.

## m6A and arrhythmia (AH)

AH is defined as the disruption of normal electrical conduction through the myocardium and can be categorized based on factors such as the ventricular rate, origin, transmission mode, and associated symptoms. Atrial fibrillation and ventricular fibrillation are the most common types of AHs, both of which are associated with an increased risk of mortality.^86^^,^^87^ The mechanisms underlying AHs involve various abnormalities at the molecular and cellular levels during the cardiac electrophysiological processes. These abnormalities include changes in ion channel function, disturbances in signaling pathways, irregularities in intracellular calcium handling, genetic mutations, and alterations in cellular architecture.^88^, ^89^, ^90^, ^91^ Notably, m6A may influence intracellular calcium processing, myocardial remodeling, and ion channel function, suggesting that m6A could play a significant role in AH development.

**Figure 3:**
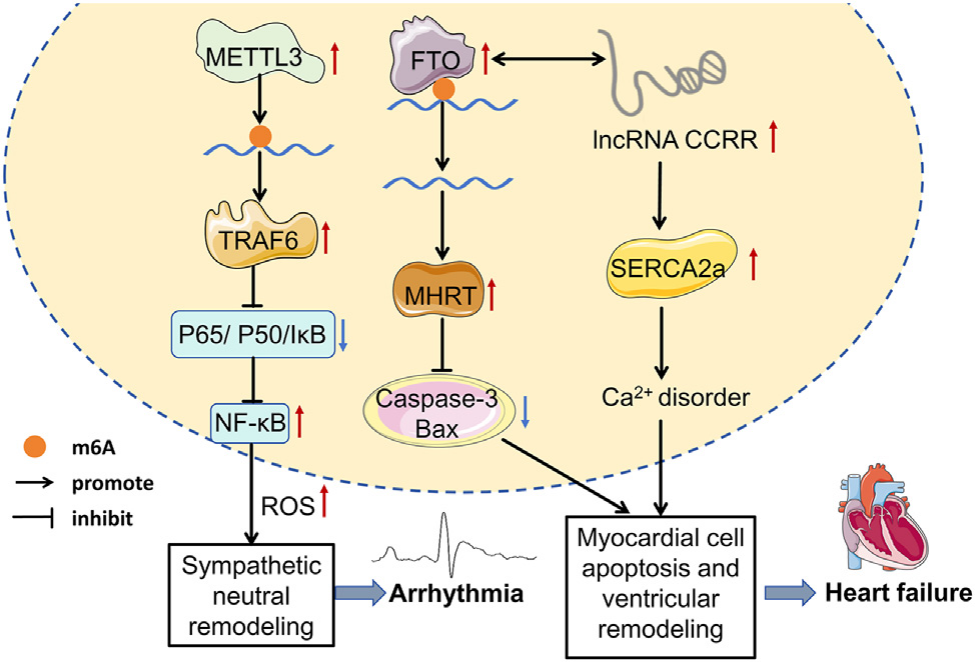
The molecular mechanism of m6A methylation in arrhythmia and HF. In arrhythmia, the overexpression of TRAF6 facilitates arrhythmia and is regulated by elevated levels of METTL3. The up-regulated FTO plays an important role in HF by increasing the expression of MHRT, thereby inhibiting myocardial cell apoptosis and ventricular remodeling. FTO also increase the expression of SERCA2a, contributing to calcium disorder and promoting the process of myocardial cell apoptosis. LncRNA CCRR and FTO reciprocally enhance each other's functions. TRAF6, tumor necrosis factor receptor-associated factor 6; FTO, Fat mass and obesity-associated protein; HF, heart failure; MHRT, myosin heavy chain associated RNAtranscript; LncRNA CCRR, long non-coding RNA cardiac conduction regulatory RNA.

Zheng et al showed that in patients with atrial fibrillation, the expression of neutrophil cytosolic factor 2 and hematopoietic cell signal transducer was significantly increased by m6A modification, which was regulated by the expression of RBM2B, IGFBP3, IGFBP5, ALKBH2, HNRNPC, and HNRNPA1B2. This alteration may affect the diversity of the immune microenvironment and signaling pathways in atrial fibrillation.^92^ Similarly, Yang et al identified three hub m6A phenotype-related genes (*RAC2*, *RELA*, and *WAS*) associated with the immune microenvironment, suggesting that m6A modification may be crucial in this context, reinforcing earlier findings.^93^ Furthermore, FTO deficiency has been shown to influence the heart rate under both resting and stress conditions through cardiac autonomic imbalance and potentially proarrhythmic remodeling, indicating a likely role of FTO in the progression of AHs.^94^ Similarly, METTL3 may also contribute to the development of AHs (Fig. 3). Reduced levels of METTL3 have been associated with the down-regulation of nerve growth factor expression, suppression of sympathetic nerve activity and inflammatory responses, and inhibition of TRAF6-dependent mitochondrial reactive oxygen species production. This change could ultimately attenuate sympathetic remodeling and constrain the TRAF6/NF-κB and TRAF6/ECSIT pathways.^95^^,^^96^

Additionally, the NF-κB signaling pathway may interact with METTL3-regulated m6A modification of Toll-like receptor 4 mRNA, thereby up-regulating Toll-like receptor 4 expression and producing pro-inflammatory cytokines IL-1β and TNF-α. This interaction can lead to sympathetic hyperactivity and ventricular AHs following myocardial infarction. However, the reduced METTL3 expression may ameliorate this pathological process. These findings suggest that the targeted modulation of METTL3 could be a potential strategy for preventing ventricular AHs after myocardial infarction.^97^

## m6A and heart failure (HF)

HF is the final common pathway for various CVDs, including ischemic cardiomyopathy (ICM) and dilated cardiomyopathy (DCM). Its etiology encompasses ventricular remodeling, inflammation, oxidative stress, mitochondrial dysfunction, and epigenetic modifications, particularly m6A modification.^98^^,^^99^ Recent studies have indicated potential dysregulation of m6A modification in HF progression, suggesting that it may play a significant role in this context (Fig. 3). Furthermore, the m6A regulators may serve as potential therapeutic targets and novel biomarkers for the diagnosis and treatment of HF.^99^, ^100^, ^101^, ^102^

m6A and its regulators play important roles in ventricular remodeling, inflammation, oxidative stress, and myocardial cell apoptosis.^98^^,^^99^^,^^102^ Yu et al demonstrated that m6A regulators, including YTHDC1, HNRNPA2B1, and heterogeneous nuclear ribonucleoprotein C, were down-regulated in ICM and DCM. This down-regulation appeared to lead to altered expression of CCL5, CXCR4, and CCL2 in ICM, and IL-6 in DCM.^100^ Zhou et al suggested that m6A abundance and its regulators may serve as promising biomarkers for the diagnosis of ICM and DCM.^101^ Additionally, ALKBH5 has been found to be up-regulated in failing hearts and hypoxic cells, which may result in reduced downstream m6A levels, suggesting that ALKBH5 could represent a valuable marker involved in the pathophysiology of HF.^103^

FTO also plays a critical role in HF by potentially attenuating ventricular remodeling and fibrosis through mechanisms related to calcium homeostasis and sarcomere dynamics. Interestingly, in contrast to ALKBH5, the expression of FTO is reportedly reduced in HF, which may lead to increased m6A levels in myosin heavy chain-associated RNA transcripts and subsequently lower its expression. This alteration can promote myocardial cell apoptosis and contribute to ventricular remodeling.^40^^,^^104^ The myosin heavy chain-associated RNA transcript is a cardiac-specific lncRNA that is thought to protect the heart from pathological hypertrophy.^105^^,^^106^ Ca homeostasis is crucial for cardiomyocyte function. Sarcoplasmic/endoplasmic reticulum Ca2+ ATPase 2a (SERCA2a), a cardiac-specific subtype of SERCA, is essential for regulating cardiomyocyte contraction and maintaining calcium homeostasis, and represents a potential therapeutic target in HF.^107^ FTO is believed to enhance SERCA2a mRNA stability in an m6A-dependent manner, and the specific knockout of FTO in cardiomyocytes has been shown to result in impaired cardiac function.^40^ Moreover, m6A modification may regulate the expression of vascular cell adhesion molecule 1, which is involved in immune cell infiltration in HF.^108^

Adriamycin, also known as doxorubicin, is a chemotherapeutic agent whose use is limited by severe cardiotoxicity that often results in AHs and chronic HF. Tu et al demonstrated that FTO regulates the binding of YTHDF1 to Toll-like receptor 4 mRNA and down-regulates Toll-like receptor 4 expression in an m6A-dependent manner. This action may inhibit doxorubicin-induced cellular pyroptosis and inflammation in HF via the TLR4/NF-κB pathway.^109^ Knockdown of circ-ZNF609 has been shown to attenuate cardiomyocyte apoptosis, reduce reactive oxygen species production, and ameliorate mitochondrial non-heme iron overload, potentially alleviating doxorubicin-induced cardiotoxicity. Additionally, Yu et al suggested that the overexpression of FTO could mitigate doxorubicin-induced cardiotoxic effects by reducing the level of m6A modification of circ-ZNF609, thus protecting the myocardium.^28^ The aging process is thought to contribute to ALKBH5-mediated cardiac m6A modifications. The elevated expression of ALKBH5 has been associated with the regulation of the DNA damage response through AT-rich interacting domain 2, which may promote doxorubicin-induced apoptosis of cardiomyocytes, thereby exacerbating doxorubicin-induced cardiac insufficiency.^110^

Other key regulators, such as METTL3 and YTHDF2, are also expressed in the heart.^36^^,^^111^ First, PIWI-interacting RNAs have been suggested to promote cardiac hypertrophy, and cardiac hypertrophy-associated PIWI-interacting RNA has been shown to regulate the m6A modification of poly (ADP-ribose) polymerase 10 mRNA in cooperation with METTL3.^112^ In cardiomyocytes, high levels of METTL3 may attenuate cardiac hypertrophy through hypermethylation of genes involved in this process, such as Rho guanine nucleotide exchange factor 3. Similarly, in the CHAPIR-METTL3-PARP10-NFATC4 signaling axis, the activity of METTL3 is reduced, which may promote cardiac hypertrophy.^113^^,^^114^

However, the accumulation of collagen proteins is considered a contributing factor in cardiac fibrosis, and high levels of METTL3 may lead to this accumulation by promoting the activation of TGFβ-related pathways. This effect appears to be opposite to the previously mentioned outcomes, indicating the need for a closer examination of the primary role of METTL3 in this process and highlighting an inherent contradiction.^115^^,^^116^ Moreover, METTL3 may participate in autophagy, which is a critical factor in cell apoptosis. The knockout of METTL3 has been associated with elevated autophagic flux, as indicated by an increased ratio of microtubule-associated protein light chain 3II to microtubule-associated protein light chain 3I.^117^ Additionally, the relationship between METTL3 and M1 macrophage polarization may play a role in HF through the regulation of inflammation, as discussed in the chapter on CADs.^61^

Furthermore, the increased expression of YTHDF2 has been observed in cardiac hypertrophy, which recognizes the m6A modification of myosin heavy chain 7 mRNA, thereby promoting its degradation and potentially alleviating myocardial hypertrophy.^118^ In addition to interacting with myosin heavy chain 7, YTHDF2 may also act downstream of myocardial infarction-associated transcript. In this pathway, the overexpression of YTHDF2 may promote cardiac hypertrophy. Yang et al indicated that the MIAT/YTHDF2/PPARα/CPT-1a signaling pathway is critical in cardiac hypertrophy, providing new insights into the mechanisms underlying HF caused by cardiac hypertrophy.^119^ Notably, both YTHDF2 and METTL3 appear to play contradictory roles in cardiac hypertrophy, suggesting the need to analyze the roles of these regulators in specific HF pathways to better understand the mechanisms and inherent contradictions. Overall, while the overexpression of METTL3 may attenuate HF development, the specific role of YTHDF2 remains inconclusive. Although the precise mechanism of m6A involvement in HF remains unclear, the aforementioned studies offer new perspectives on the hidden mechanisms and potential treatment strategies.

## m6A and ischemic reperfusion (I/R) injury

**Figure 4:**
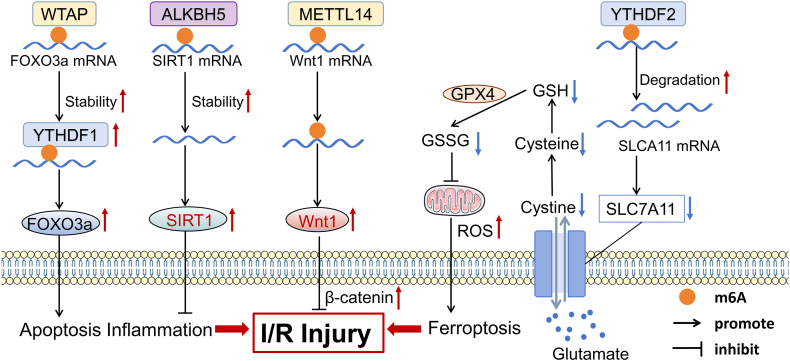
The molecular mechanism of m6A methylation in I/R injury. WTAP and YTHDF1 targets FOXO3a, promoting apoptotic inflammation. The overexpression of ALKBH5 enhances the stability of SIRT1 mRNA in an m6A-dependent manner, offering protection against I/R-induced apoptosis in cardiomyocytes. Furthermore, METTL14 regulates Wnt1 mRNA and enhances its expression, forming the β-catenin protein and inhibiting the I/R injury process. In I/R injury, YTHDF2 targets SLC7A11 mRNA to promote its degradation, thereby exacerbating iron overload and cardiac ferroptosis. I/R, Ischemic reperfusion; WTAP, Wilms' tumor 1-associating protein; FOXO3a, forkhead box O3; SIRT1, sirtuin-1; SLC7A11, solute carrier family 7, membrane 11.

Myocardial I/R injury in cardiomyocytes severely impairs cardiac function and ultimately leads to myocardial cell death following coronary recanalization, with mitochondrial dysfunction induced by oxidative stress being a key mechanism.^120^ Emerging evidence suggests that pyroptosis, autophagy, and ferroptosis may play important roles in I/R injury.^121^ Wang et al suggested that m6A modification might be an important factor in ferroptosis, potentially influenced by the imbalance between lipid reactive oxygen species and the detoxification processes of lipid hydroperoxides.^122^^,^^123^ Solute carrier family 7 member 11, which is associated with ferroptosis, may play a role in this process, as its inhibition has been linked to adverse effects on cardiac function and an increase in infarct size.^123^ According to Pang et al, YTHDF2 recognizes m6A modification sites in solute carrier family 7 member 11 mRNA, potentially leading to its degradation (Fig. 4). This mechanism may facilitate I/R injury and ferroptosis.

In contrast, the down-regulation of YTHDF2 appears to alleviate or reverse this injury.^121^ However, it is important to note that the cooperation with various downstream factors could produce different effects. The presence of YTHDF2 suggests that the expression of BCL2 interacting protein 3 may be enhanced. Notably, the up-regulation of BCL2 interacting protein 3 could potentially offset the inhibitory effects of YTHDF2 overexpression, ultimately leading to a reduction in I/R injury.^124^

Moreover, in agreement with previous studies, METTL3 appears to play a critical role in inflammation, autophagy, and the cellular stress response.^117^ Song et al suggested that METTL3 may attenuate I/R injury in the liver by inducing the expression of heme oxygenase HO-1 in an m6A-dependent manner. These findings suggest a potential treatment strategy for I/R injury in the heart.^125^ Interestingly, evidence indicates that METTL3 enhances the expression of miR-143-3p and lncRNA-small nucleolar RNA host gene 8 in cardiac tissues. It also seems to regulate the transcription of protein kinase C epsilon and δ-aminolevulinic acid synthase 2 by promoting the binding of DGCR8 to pri-miR-143-3p and the attachment of polypyrimidine tract binding protein 1 to lncRNA-small nucleolar RNA host gene 8 through m6A modification, which may exacerbate myocardial pyroptosis and I/R injury.^37^^,^^126^ Autophagy also appears to critically influence I/R injury, and METTL3 may participate in this process by down-regulating the transcription factor EB. However, the increased levels of METTL3 have been associated with reduced expression of the transcription factor EB and diminished autophagic flux, potentially promoting heart I/R injury. This phenomenon can be reversed by ALKBH5, which has an opposing effect to that observed in the liver.^117^^,^^127^ The overexpression of ALKBH5 enhances the stability of sirtuin 1 mRNA and tripartite motif-containing 72 mRNA in an m6A-dependent manner, which may offer protection against I/R-induced apoptosis in cardiomyocytes. This highlights the crucial role of ALKBH5 in cardiomyocyte apoptosis and illustrates the important regulatory influence of m6A methylation on ischemic heart diseases.^128^^,^^129^ Interestingly, Zhao et al discovered that the overexpression of ALKBH5 in hypoxic models may negatively affect peripheral blood flow recovery and hinder angiogenesis in hypoxic cardiac microvascular endothelial cells. This effect appears to be mediated by the regulation of WNT5A expression and mRNA stability in an m6A-dependent manner. Additional studies have suggested that therapeutic targeting of ALKBH5 might alleviate apoptosis in hypoxia/reoxygenation-induced H9C2 cells and neonatal rat cardiomyocytes.^45^ These findings imply that ALKBH5 could play diverse roles in hypoxia and I/R injury.^41^ Overall, although METTL3 may promote the development of I/R damage, ALKBH5 may reverse this process.

However, the role of METTL14 in I/R injury remains unclear. Pang et al indicated that a reduction in METTL14 expression significantly increased heart infarction size and lactate dehydrogenase release, and exacerbated post-I/R cardiac dysfunction. Conversely, its overexpression seemed to reverse the declining trend of Wnt family member 1 protein and β-catenin in an m6A-dependent manner (Fig. 4), which appeared to alleviate cardiac I/R damage, showing cardioprotective effects.^130^ In contrast, Wu et al revealed that METTL14 may participate in I/R injury via the Akt/mTOR signaling pathway, acting as a negative regulator. Knockdown of METTL14 reportedly inhibited I/R-induced oxidative stress and the secretion of inflammatory factors by activating the Akt/mTOR pathway, thereby reducing myocardial apoptosis and necrosis associated with I/R.^131^ Another methyltransferase of interest, WTAP, has been suggested to contribute to I/R injury via the WTAP/YTHDF1/m6A/FOXO3a axis and modification of ATF4 mRNA (Fig. 4).^132^^,^^133^ Additionally, thioredoxin-interacting protein, an essential alpha-arrestin protein, is considered crucial for maintaining cellular redox homeostasis and is involved in biological processes and pathological conditions related to post-ischemic HF.^134^ Yin et al demonstrated that the regulatory axis involving WTAP and thioredoxin-interacting protein plays a significant role in post-ischemic HF, driving the m6A modification of thioredoxin-interacting protein mRNA and enhancing its abundance.^135^

Furthermore, research has suggested that the elderly patients may be more susceptible to ischemic injury. Su et al indicated that the age-related down-regulation of METTL3 might be mitigated by decreased FTO levels during I/R injury, suggesting that FTO plays a significant role in this process.^136^ Similarly, Ke et al and Sun et al reported that FTO was down-regulated in I/R-induced cardiomyocytes and that the overexpression of FTO enhanced the stability of Yes-associated protein 1 (YAP1) mRNA. This stabilization appeared to inhibit NLRP3-mediated pyroptosis and degradation of β-catenin induced by CBL, ultimately reducing I/R-induced cardiomyocyte apoptosis and inflammation.^137^^,^^138^

These findings suggest that m6A plays a significant role in myocardial I/R injury and may offer potential therapeutic strategies. However, it is essential to note that no single factor functions in isolation; therefore, the downstream interactions should be carefully evaluated to ensure appropriate application.

## m6A and other cardiovascular diseases

In aneurysms, m6A modifications are crucial for regulating ferroptosis in vascular smooth muscle cells. In mice with thoracic aortic aneurysms, an increase in iron death within the thoracic aorta has been observed alongside the up-regulation of METTL14 levels. METTL14 knockdown inhibits the progression of thoracic aortic aneurysms by reducing iron death in vascular smooth muscle cells. This process may be linked to the m6A regulation of ACSL4 mRNA stability, which involves METTL14 modification and recognition by IGF2BP2.^139^ Additionally, patients with abdominal aortic aneurysms exhibiting higher m6A levels are more likely to experience aneurysm rupture, which is closely associated with YTHDF3 levels.^140^ In addition, FTO expression significantly promotes the phenotypic switching of vascular smooth muscle cells and aortic dissecting aneurysms by regulating the m6A levels of Krüppel-like factor 5 and phosphorylated glycogen synthase kinase 3β.^141^

m6A also plays an important role in vascular calcification. ALKBH1 demethylates DNA m6A modifications, which promotes the binding of octamer-binding transcription factor 4 (Oct4) to the promoter of bone morphogenetic protein 2, thereby activating bone morphogenetic protein 2 transcription. This process contributes to the osteogenic reprogramming of vascular smooth muscle cells and facilitates subsequent vascular calcification progression.^142^ METTL14 expression is elevated in calcific arteries and human aortic smooth muscle cells induced by indoxyl sulfate, increasing m6A levels in RNA and reducing vascular repair function. The reduced METTL14 expression in human aortic smooth muscle cell calcification models may decrease calcification and enhance vascular repair function.^143^

## Conclusions and future perspectives

m6A is the most abundant and conserved epigenetic modification of eukaryotic RNAs, and is closely associated with the transcription, cleavage, translation, and degradation of target mRNA. The regulation of m6A RNA modification occurs through m6A methyltransferases and demethylases, which are subsequently recognized by the m6A binding proteins YTHDF1/2/3, YTHDC1/2, IGF2BP1/2/3, and HNRNPA2B1, thereby facilitating the interaction of target mRNAs within cells. First described in the 1970s, m6A has emerged as a prominent RNA modification and a critical regulator of various biological processes and diseases, including cancers and CVDs.

m6A studies related to cancers are the most advanced and prevalent.^144^ In cancers, m6A levels are related to DNA damage and repair (genomic instability), cell proliferation and metastasis, the immune microenvironment, and immunosuppression. m6A modification regulates the stability of various oncogene mRNA and promotes mature mRNA decay, which inhibits cancer progression. Therefore, the key regulators of m6A modification are potentially important molecular targets in cancer therapy. In addition, m6A is an emerging cancer drug target and an important biomarker that may help in the screening of precancerous lesions, the early diagnosis of cancer, and immunotherapy, which may play an important role in further understanding antitumor drug resistance and other aspects.^145^, ^146^, ^147^, ^148^, ^149^, ^150^, ^151^, ^152^, ^153^

Similar to cancer, the prevalence of CVDs remains high globally and poses a significant threat to human health. m6A modification influences various physiological processes in cardiomyocytes, including proliferation, apoptosis, and metabolic activities, all of which are crucial for cardiovascular health. These findings may contribute to the development of early diagnostic methods and improve therapeutic interventions for the treatment of HF, providing hope for countless patients. This uniqueness underscores the importance of gaining a deeper understanding of the biological mechanisms of m6A modification in CVDs while also providing new directions and insights for clinical research.

**Figure 5:**
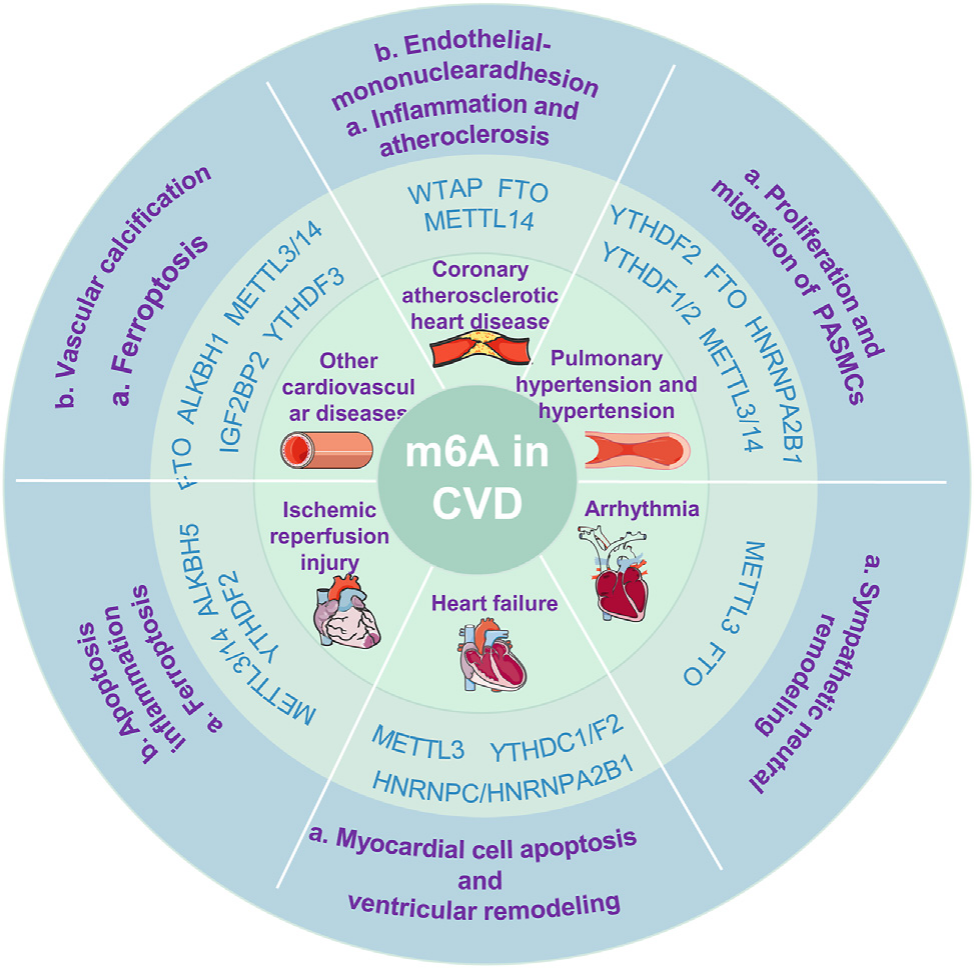
Critical regulators of m6A in cardiovascular disease (CVD): A summary of this passage. The regulators of m6A in coronary atherosclerotic heart disease, pulmonary hypertension, hypertension, arrhythmia, heart failure, and ischemia-reperfusion injury involve complex interactions among m6A methyltransferases, demethylases, and reader proteins. This complexity underscores the potential of m6A as a target in the treatment of CVDs.

This review emphasizes the complex interactions among m6A methyltransferases, demethylases, and reader proteins, highlighting the potential of m6A as a therapeutic target for CVDs. We elucidated the critical roles of m6A methylation in various cardiovascular conditions, including CADs, PH, hypertension, AH, HF, and I/R injury (Fig. 5). However, our observations revealed that some regulatory proteins may play conflicting roles in specific diseases. For instance, Xu et al reported that the increased expression of YTHDF2 in cardiac hypertrophy interacts with the m6A modification of Myh7 mRNA, thereby alleviating myocardial hypertrophy.^118^ In contrast, Yang et al found that in the MIAT/YTHDF2/PPARα/CPT-1a signaling pathway, the overexpression of YTHDF2 exacerbates cardiac hypertrophy.^119^ These findings suggest that we should analyze the roles of these regulators not only as a collective entity but also within specific pathways in CVDs to better understand the underlying balance and mechanisms at play. Moreover, it is crucial to address key contradictions, such as the age-related down-regulation of FTO and METTL3, in the elderly patients with acute I/R injury. Gaining insight into these dynamics could significantly enhance our ability to effectively apply these findings to the diagnosis and treatment of CVDs.

In conclusion, this review aimed to synthesize the latest findings related to m6A's involvement in CVDs to uncover new therapeutic strategies for its management. However, further experimental and clinical evidence is required to confirm this possibility. Future studies should focus on elucidating the functions of m6A and uncovering the underlying mechanisms of CVDs, which represents a promising avenue for the treatment and/or diagnosis of CVDs.

## CRediT authorship contribution statement

**Hongjiao Liu:** Writing – review & editing, Writing – original draft. **Tao Song:** Writing – review & editing. **Yan Huang:** Writing – review & editing, Writing – original draft, Supervision.

## Funding

This research was funded by the 10.13039/501100001809National Natural Science Foundation of China (No. 82100331), the 10.13039/501100003819Natural Science Foundation of Hubei Province, China (No. 2023AFB797, 2023AFB806), the Knowledge Innovation Program of Wuhan-Shuguang Project (China) (No.2022020801020484), and 10.13039/501100012226Fundamental Research Funds for the Central Universities of China (No. 2042019kf0058) to Yan Huang.

## Conflict of interests

The authors declared no competing interests.
